# Development and use of a custom-designed vaginal dilator for post-surgical management in a congenital adrenal hyperplasia patient

**DOI:** 10.3389/fmed.2026.1756295

**Published:** 2026-05-25

**Authors:** Camil Castelo-Branco, Marta Camacho, Ramon Farré

**Affiliations:** 1 Service of Gynecology, Institut Clinic de Ginecologia, Obstetricia I Neonatologia, Hospital Clinic of Barcelona, University of Barcelona, Barcelona, Spain; 2 Institut d'Investigacions Biomèdiques August Pi i Sunyer (IDIBAPS), University of Barcelona, Barcelona, Spain; 3 Unit of Biophysics and Bioengineering, School of Medicine and Health Sciences, University of Barcelona, Barcelona, Spain

**Keywords:** biomedical engineered dilators, congenital adrenal hyperplasia, genioplasty, open-source, vaginal stenosis

## Abstract

We present the open-source development and application of a novel, custom-engineered vaginal dilator for the post-surgical management of vaginal stenosis in a 29-year-old patient with congenital adrenal hyperplasia. This methodology highlights a collaborative approach between surgical teams and biomedical engineers, addressing limitations in traditional dilators by designing patient-specific molds based on MRI measurements. The dilator, made of biocompatible silicone elastomer and created using a simple 3D-printing process, demonstrated superior patient comfort and compliance in this patient. The use of this innovative device could improve surgical outcomes, reduced re-stenosis rates, and enhanced patient quality of life. Additionally, the surgical approach for clitoral reduction is described, emphasizing its complexity and the importance of preserving neurovascular integrity to avoid complications such as pain or sensory loss. The case underscores the importance of tailored interventions, as well as interdisciplinary collaboration and the potential of this for broader application of novel methodologies in similar clinical contexts.

## Introduction

Congenital adrenal hyperplasia (CAH) is one of the most prevalent inherited disorders (roughly 1 out of every 10,000 births) and encompasses a group of autosomal recessive conditions caused by inactivating mutations in single enzymes essential for cortisol biosynthesis (1). Women with CAH can experience effects across several health outcomes and quality-of-life domains, reflecting both the hormonal condition itself and the impact of early-life treatments. Research consistently shows that sexual function, fertility, and urogenital health are among the most affected medical outcomes, with some women reporting pain during intercourse, reduced sexual satisfaction, or anatomical challenges related to childhood surgery. Psychosocial domains are also frequently impacted: studies describe higher rates of body-image concerns, gender-role stress, and emotional well-being difficulties, often linked to early virilization, medical interventions, and long-term monitoring. Broader quality-of-life measures —such as social relationships, self-esteem, and overall life satisfaction— tend to be somewhat lower on average compared with unaffected women, though many individuals report good functioning, especially when supported by modern multidisciplinary care (1). The most common CAH form is due to 21-hydroxylase deficiency, which is associated with genital virilization in 46XX individuals, often necessitating complex surgical interventions for the purpose of restoring normal and functional anatomy (2). Genital reconstructive surgeries are technically complex and can lead to intraoperative and postoperative complications, with variable long-term outcomes (3). According to a recent systematic review, complications such as clitoral sensitivity loss, vaginal stenosis, and difficulties with penetration remain prevalent after surgery, affecting up to 27% of cases (4), and significantly impacting long-term outcomes and quality of life (3, 5). Sexual dysfunction also remains a key concern despite some surgical advancements (3, 6).

One common complication of such surgeries that often require re-intervention is vaginal stenosis (7). Traditional vaginal dilators are often uncomfortable and poorly adapted to individual anatomy, leading to inconsistent usage and suboptimal results. This study aims to introduce a novel, custom-designed vaginal dilator as a possible solution to these challenges, while also presenting the surgical approach used in this case, highlighting the importance of tailored interventions for optimal outcomes.

## Methodology

**Figure 1:**
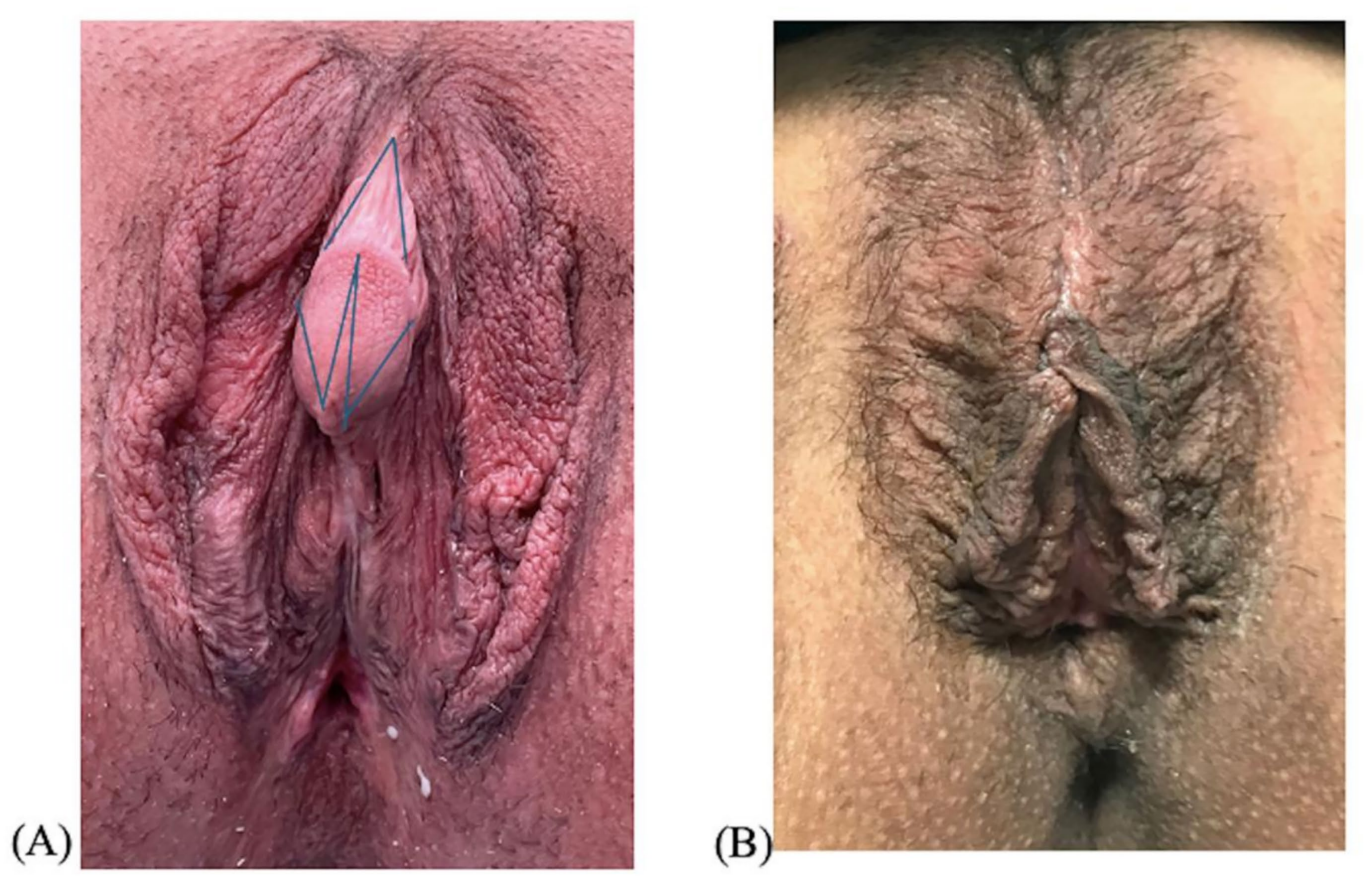
Preoperative anatomy **(A)** shows clitoromegaly, fusion of the labia minora, and a markedly narrowed vaginal introitus, consistent with virilizing anatomy. The postoperative image after 6 months **(B)** shows reduced clitoral size with preservation of neurovascular structures, restoration of separate labial anatomy, and a widened, functional vaginal opening reflecting a restored and improved vulvar configuration after surgical correction.

A 29-year-old virilized female individual (46, XX karyotype), diagnosed with CAH due to 21-hydroxylase deficiency (2) came to our institution to consider reconstructive surgery. On initial evaluation, she showed clitoromegaly, fusion of the labia minora and a stenotic vaginal orifice (Prader II, according to Andreas Prader first classification (8)), which was consistent with cases of moderate to severe virilization as previously described (7, 9). The first surgical approach included clitoral reduction through two V-W shaped (one inverted) and the creation of a clitoral hood is shown in Figure 1. We carefully preserved the neurovascular bundle, which prevented complications such as pain or loss of sensitivity often reported in similar cases (4) and we completed the opening of the vaginal introitus at the same time.

Following this initial surgery, the patient experienced re-stenosis due to inconsistent use of standard dilators as she experienced severe discomfort with the available marketed models (Vagiwell ® Kessel Medintim GmbH. 64,546 Mörfelden-Walldorf). Given the limitations experienced, we developed a custom-engineered vaginal dilator in collaboration with the Unit of Biophysics and Bioengineering at the University of Barcelona (Spain).

Written informed consent was obtained from the patient for the publication of this case including all the pictures. The study was conducted in accordance with institutional ethical guidelines.

### Design of the custom-engineered vaginal dilator

The customized dilator was designed as a cylinder with rounded bases (each a hemisphere with the same diameter as the cylinder), with diameter and length tailored to the patient’s anatomy using MRI imaging. The dilator was crafted from a silicone elastomer (Elastosil® Vario 15 crosslinked with Elastosil® Cat Vario in a 10:1 ratio, Wacker Chemie AG, Munich, Germany), a biocompatible material commonly used in biomedical applications (10). This material was chosen for its ease of preparation, as it does not require degassing (a process using vacuum pressure to eliminate air bubbles in the preparation), cures at room temperature in 3 h, and retains low hardness (nominal 15 Shore A), making it ideal for vaginal use. Indeed, a Shore A hardness of 15 corresponds to a very soft elastomeric material, comparable to the consistency of a soft eraser or firm gelatin (see the Figure in file “Dilator stiffness characterization” in Supplementary material). Additionally, the external side of the dilator is equipped with a thread loop to facilitate easy removal by the patient.

The fabrication process involved mixing 100 g of silicone elastomer with 10 g of crosslinker. The silicone crosslinker is a chemical agent that molecularly connects linear silicone polymer chains into a 3D network, transforming liquid or paste silicone into a cured, rubbery elastomer. These components are translucent, as will be the resulting dilator. This mixture was manually stirred for 3 min (e.g., with a disposable pipette tip), and poured directly (since the crosslink had already started), into a custom 3D-printed mold, designed using polylactic acid (PLA) as printing ink, in a conventional 3D printer. The dilator dimensions can be easily modified using free 3D-printer software (e.g., UltiMaker Cura®).

**Figure 2:**
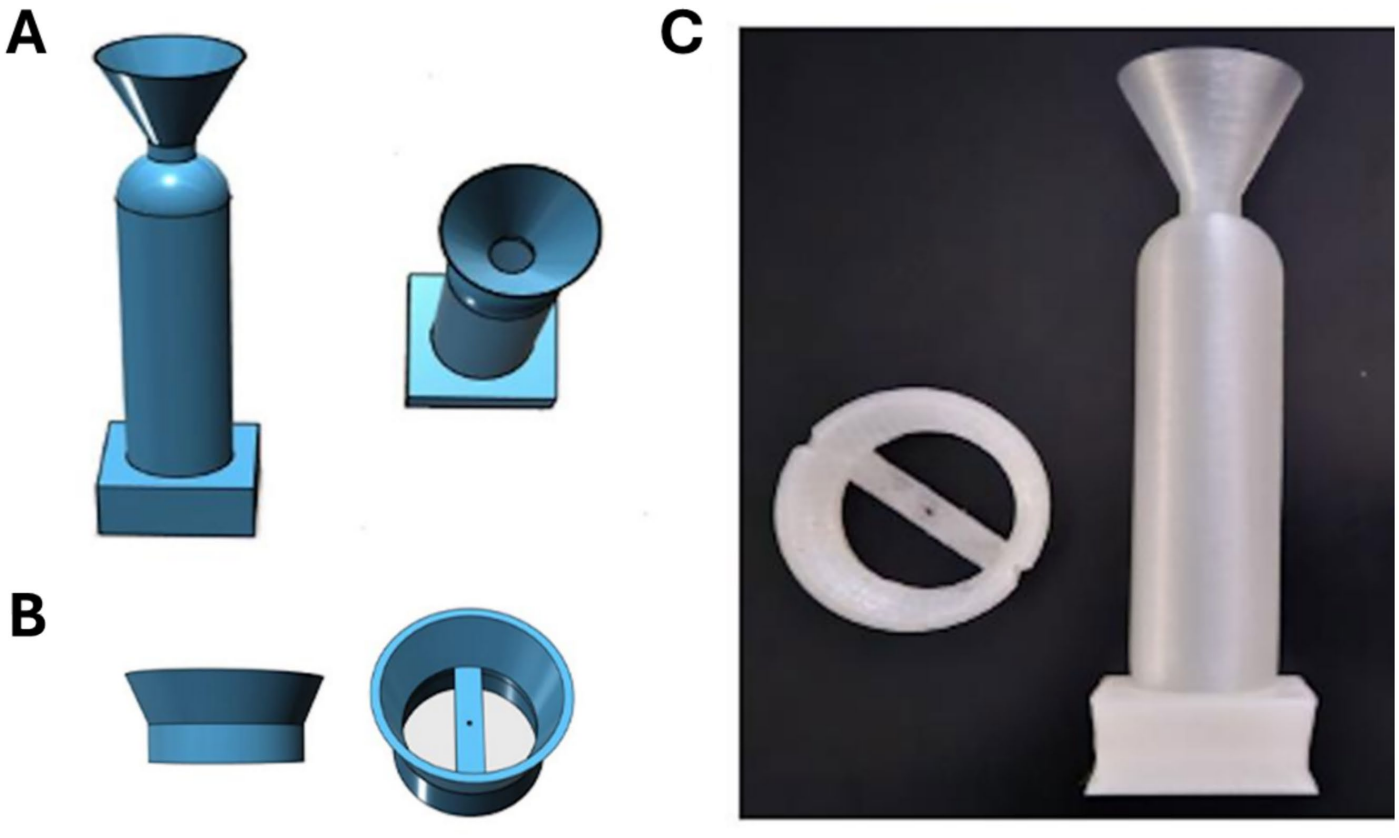
It shows 3D-printed pieces for molding the customized vaginal dilator. **(A)** Sketch of the dilator mold. **(B)** Sketch of the piece to center the thread section within the mold. **(C)** Printed pieces to observe their actual appearance and relative sizes. These pieces were 3D-printed using a 0.25 mm nozzle with a 0.1 mm layer height setting.

The molding set consists of two pieces as seen in Figure 2. A disposable mold with a funnel for introducing the silicone mixture (Figure 2A), and a reusable piece that aligns the external thread loop along the cylinder axis (Figure 2B). Figure 2C shows the actual appearance and relative size of both pieces. The open-source standard (. STL format.) files allowing the preparation of the required pieces by any conventional low-cost (1000–1,500 US$) 3D printer can be found in Supplementary material.

For the retrieval loop we used a polypropylene monofilament (Metric 4, Surgipro®, Medtronic, Madrid, Spain). We selected this surgical thread among others because we verified that it presents less reduction in traction force failure after conventional sterilization with ethylene oxide. A detailed graphical description of how the mold is prepared can be shown in file “Mold preparation & final coating” in Supplementary material.

**Figure 3:**
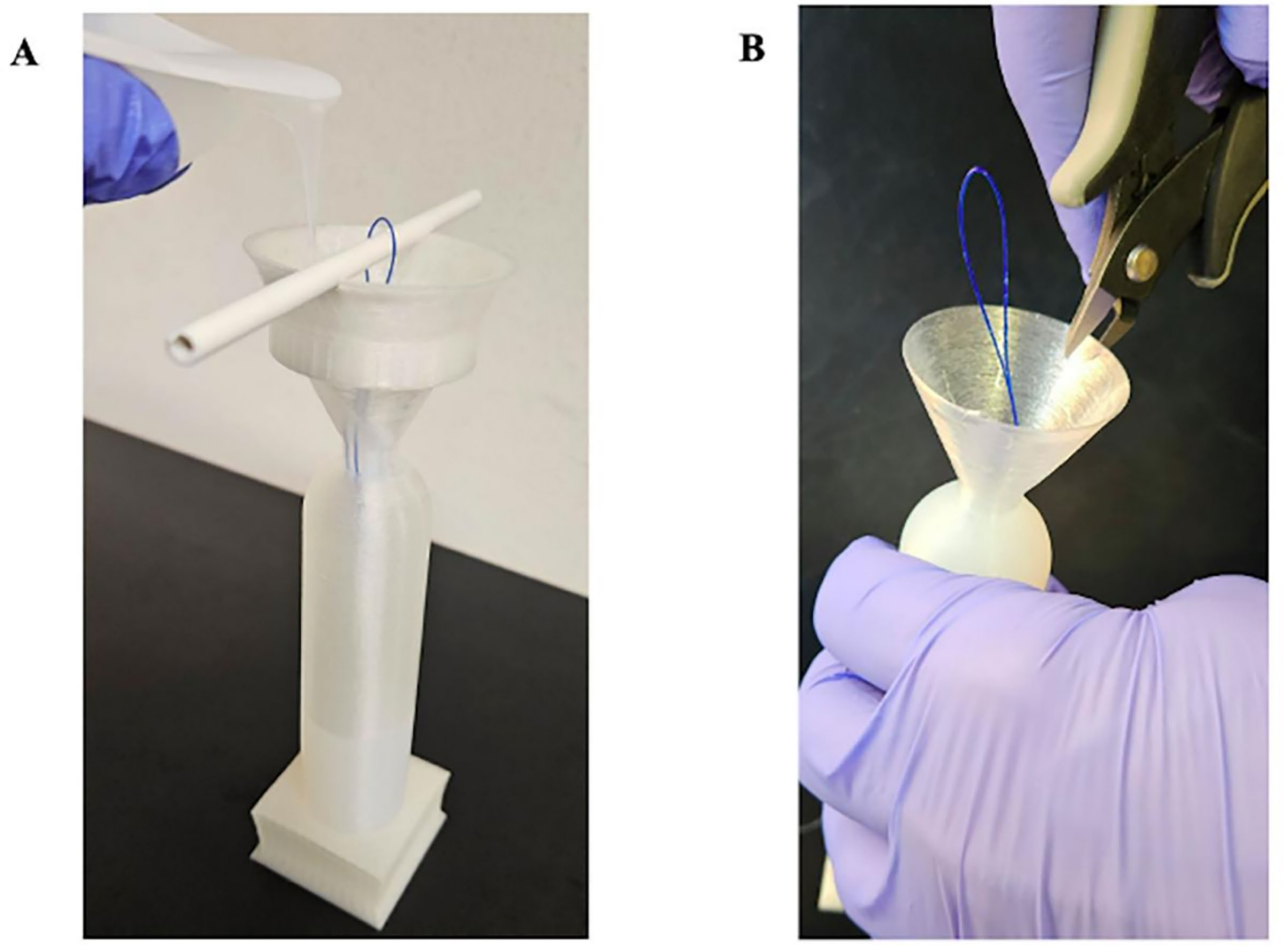
Molding and demolding the customized vaginal dilator. **(A)** The preparation mixture is poured into the mold. **(B)** After curing, the mold walls are cut to obtain the dilator.

Figure 3A shows how the recently mixed components are placed into the mold. The mixture is carefully poured close to the lateral side of the funnel. Pouring should be slow enough to avoid accumulation of components in the narrow section of the funnel. The translucent walls of the mold allow observation of the rising mixture surface. If poured smoothly, no air bubbles will form within the mold (thus, eliminating the need for degassing). The mold should be filled until the surface of the mixture reaches the rounded top of the dilator. A conventional syringe and needle (e.g., for intramuscular injection) can be used to remove or add any small amount of mixture to adjust its level to the rounded tip of the dilator. After curing at room temperature for 4 h, the very thin wall (0.75 mm) of the mold is easily cut to extract the dilator (Figure 3B). Subsequently, as graphically shown in Supplementary material, the thread at the bottom of the dilator is cut flush, and the dilator is then dipped once in recently prepared mix of components (silicone plus crosslinker) and allowed to cure (for 2 h) while hold by the retrieval loop (Figures J–N in file “Mold preparation & final coating” in Supplementary material). This procedure ensures that the area of the thread cut at the dilator’s bottom is uniformly covered by silicone and the whole dilator has a very smooth surface (regardless of the resolution at which the mold was 3D-printed). Finally, the dilator is washed with surgical soap, sterilized with conventional ethylene oxide and made ready for use.

The retail cost of the 3D-printer filament (50 g) for the mold and the silicone elastomer (100 g) and crosslinker (10 g) required to produce one dilator amounts ≈ 10 US$.

## Results

The patient’s sexual function and satisfaction were restored according FSFI (female sexual function index) and CSFQ-F-C (changes in sexual functioning questionarie-female clinical version) criteria, showing both questionnaires clinical improvement and highlighting the efficacy of a tailored surgical approach and the successful integration of the surgical and engineering innovations (see Supplementary Table 1).

**Figure 4:**
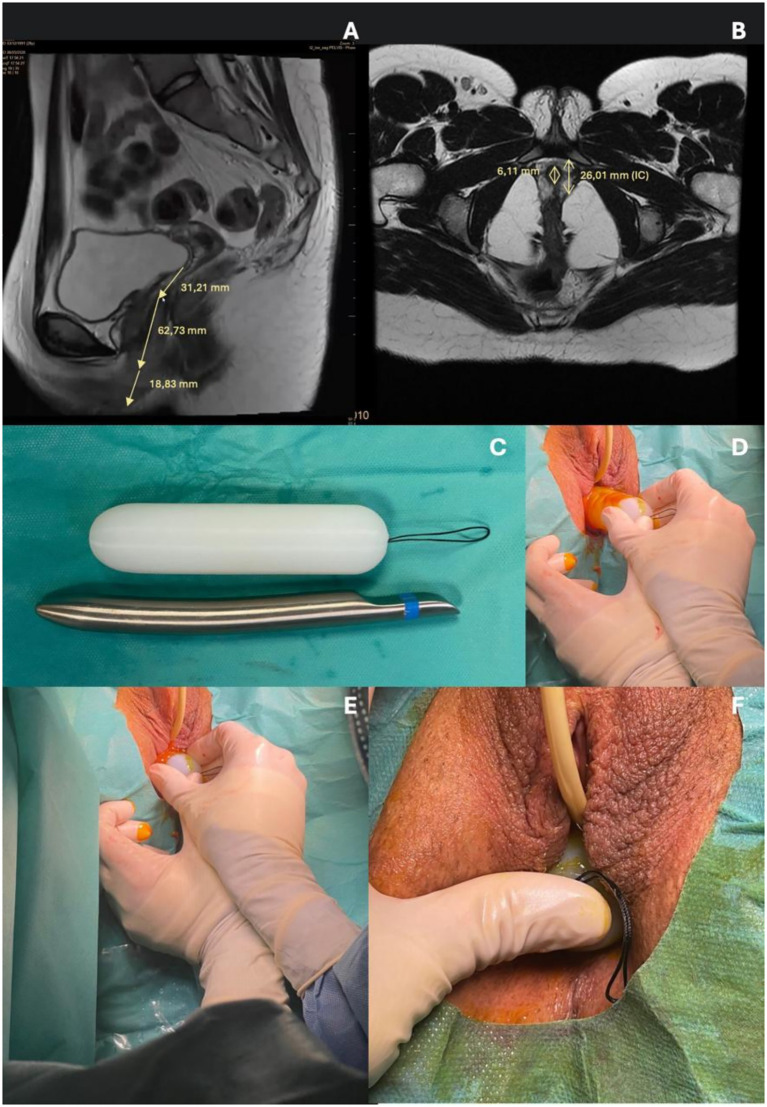
**(A,B)** Show the measurements made by magnetic resonance in order to tailor the design of the dilator. **(C)** Shows the final result of the biomedical engineered dilator. **(D–F)** Show the complete introduction of the designed dilatator after surgery.

More importantly, the custom-designed dilator significantly improved the surgical outcome in this patient. The patient reported high levels of comfort and adherence to the prescribed regimen (VAS 9/10 compared to VAS 6/10 when referring to the non-individualized marketed dilator), with no re-stenosis observed at 12- and 24-month follow-ups. As seen in Figure 4, the customed vaginal dilator in this patient measured 26 mm in diameter and 12 cm in length according to her end-to-end vaginal distance measured by magnetic resonance imaging (Figures 4A,B), and was equipped with the polypropylene monofilament previously described (Figure 4C). The dilator was inserted immediately post-surgery and used daily to prevent the vaginal introitus from closing (Figures 4D–F).

Compared to conventional surgical procedures and dilators, this methodology provided superior anatomical fit and patient compliance, leading to improved overall outcomes (Table 1).

**Table 1:** Comparative overview of standard surgical management for virilizing congenital adrenal hyperplasia versus the approach implemented in the present case.

Aspect	Standard procedure (typical approach in CAH reconstruction)	This case (documented approach)
Clitoral surgery technique	Clitoral reduction often uses recession or nerve-sparing reduction; techniques vary, and neurovascular preservation is recommended but not always consistently achieved in older literature.	Two V-W shaped incisions (one inverted) with explicit preservation of the neurovascular bundle to avoid pain or loss of sensitivity.
Vaginal introitus management	Standard vaginoplasty may include introitoplasty; stenosis is a known complication, often requiring postoperative dilation.	Opening of the vaginal introitus performed simultaneously with clitoral surgery.
Postoperative dilation strategy	Commercial dilators are typically used; discomfort and poor adherence are common, and re-stenosis is a frequent complication.	A custom-engineered dilator was created due to discomfort with standard models, improving adherence and preventing re-stenosis.
Dilator material	Commercial dilators are usually rigid medical-grade plastics or silicone with fixed dimensions.	Soft silicone elastomer (15 Shore A), tailored to patient anatomy; chosen for softness and biocompatibility.
Dilator dimensions	Standard dilators come in preset sizes, not individualized.	MRI-based customization: 26 mm diameter, 12 cm length.
Dilator fabrication	Manufactured commercially; no customization; no patient-specific molds.	3D-printed mold, custom geometry, silicone poured and cured at room temperature; low-cost production (~10 USD).
Retrieval mechanism	Standard dilators typically have no retrieval loop.	Polypropylene monofilament retrieval loop, selected for stability after sterilization.
Surface finishing	Commercial dilators have industrial finishing; not customizable.	Hand-finished by dipping in fresh silicone to ensure a smooth surface and cover the thread insertion point.
Sterilization	Commercial devices are sterilized industrially; reusable models may not be sterilizable.	Ethylene oxide sterilization performed after fabrication.
Outcome	Re-stenosis is common; adherence varies; discomfort often reported.	High comfort (VAS 9/10), no re-stenosis at 12 and 24 months, improved sexual function scores.

## Discussion

Most girls with classic CAH who are born with significant virilization historically underwent early genital surgery, and in many centers this meant the majority of affected infants received procedures such as clitoroplasty or vaginoplasty. In recent years, however, clinical practice has shifted toward delaying, individualizing, or avoiding surgery unless medically necessary, so the proportion of patients undergoing surgical repair is now lower and highly variable across countries and institutions. These trends reflect evolving ethical standards, increased emphasis on shared decision-making, and recognition of long-term outcome uncertainties.

Despite these significant changes in medical and surgical management over the past two decades (11), including adoption of single-stage surgeries for genital anomalies, long-term outcomes often remain unsatisfactory (12). Complications such as vaginal stenosis, impaired sexual function, and dissatisfaction with cosmetic results continue to be prevalent challenges, as highlighted in retrospective studies (4). This underscores the importance of fostering global collaboration among experts to share clinical experiences and improve management strategies for CAH patients.

Commercially available vaginal dilators often present significant disadvantages, including inadequate dimensions that fail to fit the patient’s anatomy, frequently being excessively long or rigid. Moreover, their shape is not always appropriate for patients with previous genital reconstructive surgery, as they do not accommodate to potential anatomical variations resulting from genitoplasty. These limitations can contribute to patient discomfort, poor adherence, and ultimately, suboptimal surgical outcomes.

Our case highlights the value of a tailored approach that integrates surgical techniques with innovative biomedical solutions. The use of custom-designed vaginal dilator tailored to our patient’s anatomy represents a significant advancement over standard models, reducing complications and potentially enhancing patient satisfaction. In our case, the patient effectively reported that the custom-designed dilator was significantly more comfortable and preferable compared to the standard model prescribed initially. The methodology described for preparing the dilator is straightforward, requiring minimal training and preparation time, with the entire process - from mixing to extraction - taking less than 20 min. The open-source mold design (Supplementary material), allows for easy replication and adaptation, making it highly versatile for patient-specific needs.

While the creation of this custom-made dilator adapted to the patient’s anatomy is novel and serves as proof of concept, we must acknowledge its limited generalizability, as we are presenting only one case and one specific pathology. Its use in other pathologies, such as Rokitansky or Morris syndromes, which require the creation of neovaginas, and its testing in a larger number of cases would help expand its application and to demonstrate its usefulness and safety. Moreover, this interdisciplinary approach could have potential applications requiring vaginal dilators such as post-radiation therapy or gender-affirming surgeries.

This case has important clinical implications. First, meticulous preservation of the clitoral neurovascular bundle during reduction helps avoid postoperative sensory loss and pain, complications that have historically affected patient satisfaction. The positive postoperative sexual function scores reinforce the importance of nerve sparing techniques in CAH reconstructive surgery. Second, the importance of individualized postoperative dilation since standard dilators often fail to accommodate anatomical variability, contributing to discomfort and poor adherence. Third, the integration of low-cost engineering solutions using a custom 3D printed mold and biocompatible silicone. This approach makes personalization accessible in routine clinical practice, especially in settings where commercial options are limited or poorly tolerated. Fourth, we enhanced long term outcomes through personalization performing tailored surgery and developing a customized dilator that resulted in sustained anatomical patency and improved sexual function. This suggests that personalization—rather than reliance on standardized tools—may be key to reducing restenosis rates and improving quality of life in CAH patients undergoing genital reconstruction. And finally, because the design files are open-source and the materials inexpensive, this methodology can be replicated in other centers. It offers a scalable model for integrating engineering innovation into reconstructive gynecology, particularly for conditions where postoperative dilation is essential.

Future research should explore the applicability of this methodology, while also evaluating long-term outcomes to further optimize care for these complex cases. By combining clinical expertise with innovative technologies, significant advancements can be made in improving both functional and psychosocial outcomes for CAH patients.

## Conclusion

Genital surgery in patients with CAH is technically complex and remains controversial; it is difficult to assess optimal time of surgery, type of surgery considering issues such as potential poor long-term somatic and psychological effects and complications such as pain, incontinence and appearance of fistulas (7). The custom biomedical engineered vaginal dilator designed specifically for this patient was essential in obtaining good surgical outcomes, preventing re-stenosis, and improving the patient’s overall quality of life. The collaboration between surgical teams and biomedical engineering departments, as seen in this case, highlights the importance of interdisciplinary innovation in improving patient care (13).

## Data Availability

The raw data supporting the conclusions of this article will be made available by the authors, without undue reservation.
