# Supplementary material for: Development and use of a custom-designed vaginal dilator for post-surgical management in a congenital adrenal hyperplasia patient
Source: Front Med (Lausanne). 2026 May 25;13:1756295. doi: 10.3389/fmed.2026.1756295 (PMC13243258; doi:10.3389/fmed.2026.1756295)
Supplement: Supplementary file 1 [file Data_Sheet_1.ZIP › Supplementary files/DILATOR STIFFNESS CHARACTERIZATION.pdf]

DILATOR STIFFNESS CHARACTERIZATION

Soft polymer stiffness is usually characterized using a Shore (A or B) index based on indenting the material with specific probes. However, this technical index does not provide a direct intuitive measure of how flexible a dilator made with the polymer is. A more intuitive test is to use the classic basic theory describing the maximum deflection Δ of a cylindrical beam when a force F is applied at its center, as shown in the Figure. Δ depends on the beam diameter D, the lenght L between the subjection points and the Young's modulus E of the material.

To illustrate the high flexibility of the dilators we made, a force was applied at the center of a dilator (D=3 cm, L=10 cm) and Δ was measured. A relatively low force of F = 0.5 kg induced a deflection Δ=11 mm. This actual measurement indicates that the Young's modulus E of the dilator material is 0.24 MPa, a value characterizing extremely soft and compliant materials, within the range of biological soft tissues.

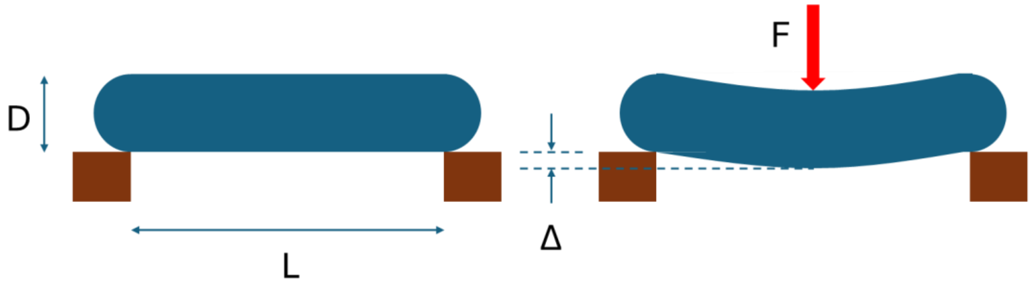

$$\Delta = \frac{4 \cdot F \cdot L^3}{3 \cdot \pi \cdot E \cdot D^4}$$

$$\left.\begin{array}{l} D = 3\text{ cm} \\ L = 10\text{ cm} \\ F = 500\text{ g} \end{array}\right\} \Delta = 11\text{ mm}$$
