# Supplementary material for: Development and use of a custom-designed vaginal dilator for post-surgical management in a congenital adrenal hyperplasia patient
Source: Front Med (Lausanne). 2026 May 25;13:1756295. doi: 10.3389/fmed.2026.1756295 (PMC13243258; doi:10.3389/fmed.2026.1756295)

MOLD PREPARATION

From the 3D printed mold (A), the center of its base is marked, and a 1 mm diameter hole is drilled (B). The thread is passed from the outside of the base to the mold inside (C) and then it is extracted through the upper part of the mold (cone) with the help of tweezers (D). A double knot is made to close the loop (E).

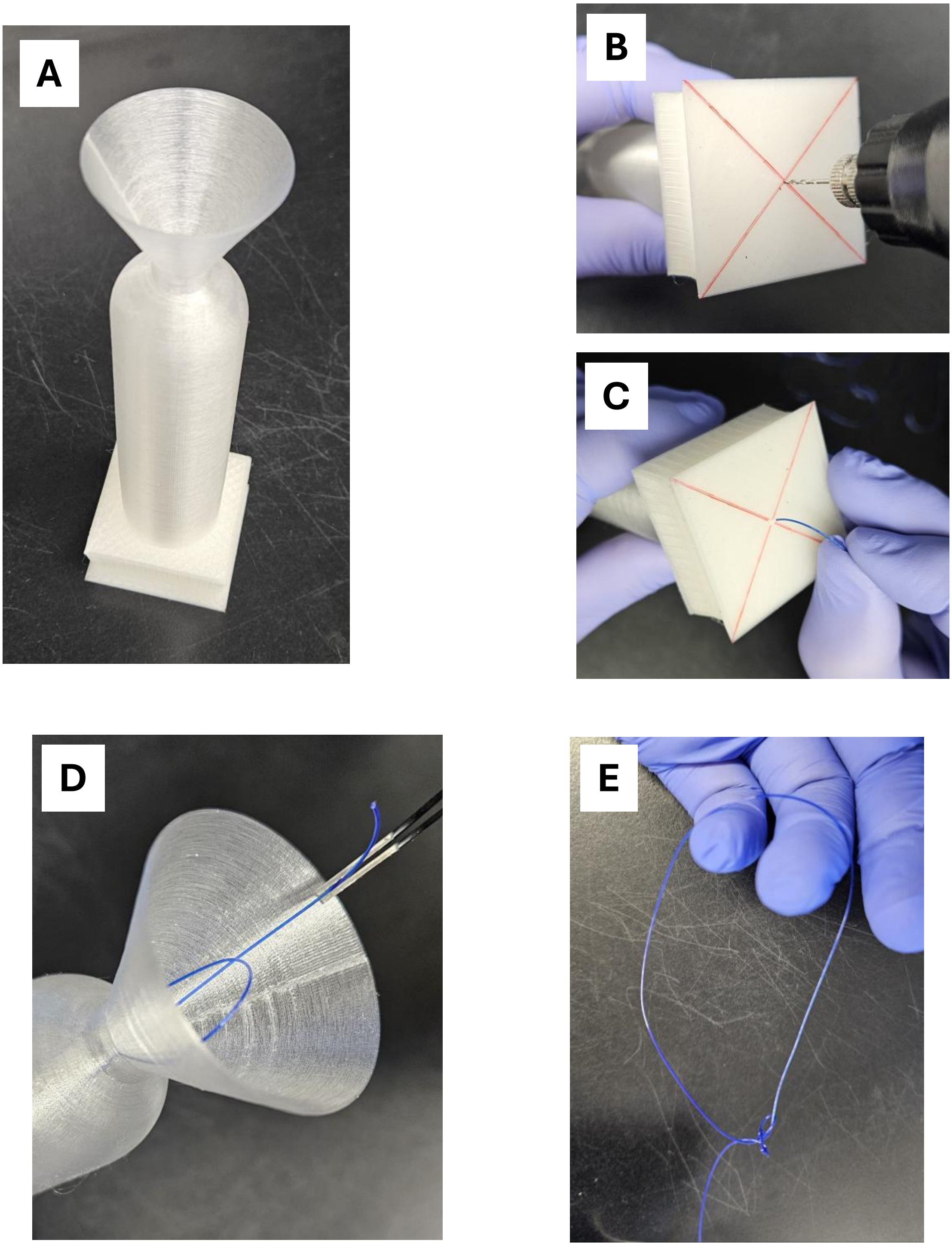

The cylindrical piece is placed inside the mold and the thread is tightened underneath the mold so that the loop has the correct size (F). To keep the cable taut, it is secured with adhesive tape to the external base of the mold (G). This adhesive tape, in addition to tightening the cable, acts as a seal to prevent that, when it will be placed into the mold, the liquid silicone compound leaks out through the hole in the base of the mold (H). Through the translucent walls of the mold, Figure (I) shows that the cable is taut and that the internal double knot (arrow) is in a centered area in the mold. Its exact position is however not relevant provided that it is not close to the upper or lower parts of the mold.

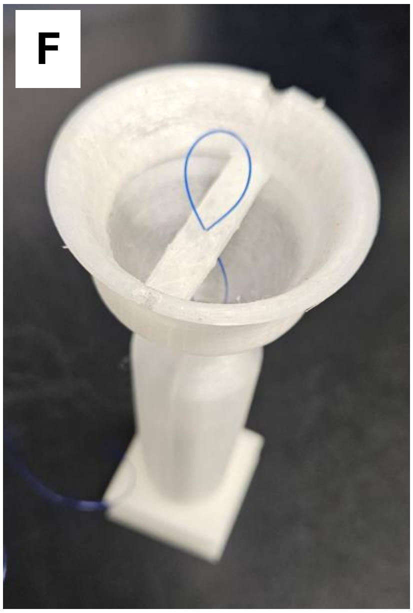

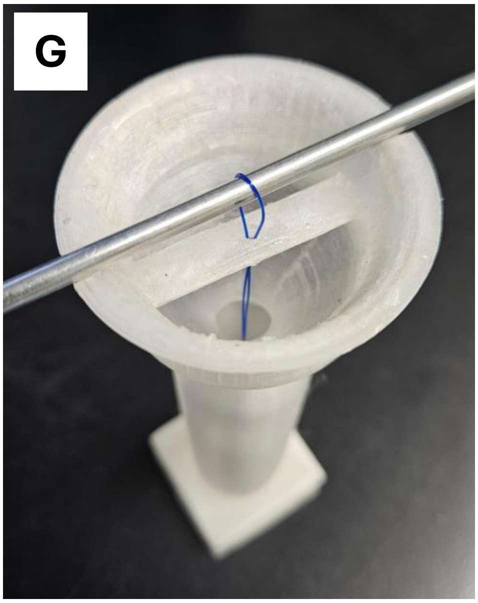

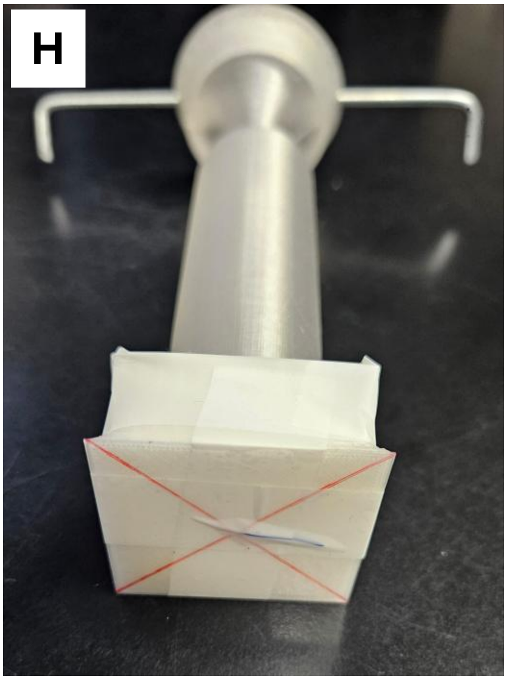

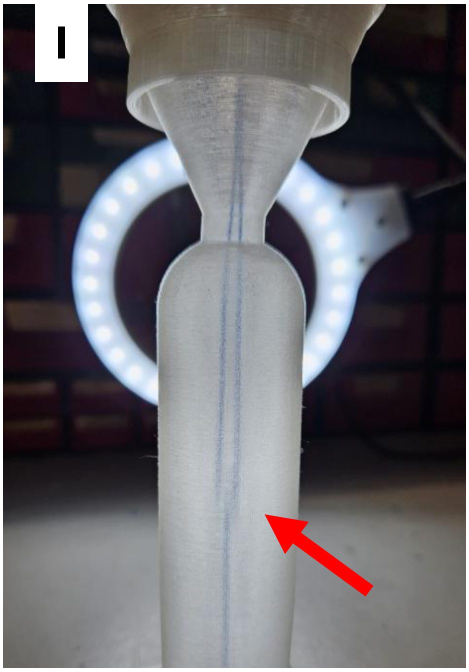

FINAL COATING

Once the dilator has been extracted from the mold, the thread leaving through the lower part of the dilator is cut flush (J, K), and the dilator is then dipped once in recently prepared mix of components (silicone plus crosslinker) (L) and allowed to cure for 2 h while hold by the retrieval loop (M). The final surface of the whole dilator is very smooth including the bottom part where the exceeding thread was cut (N).

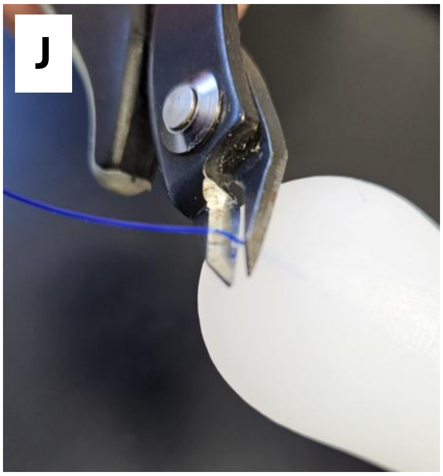

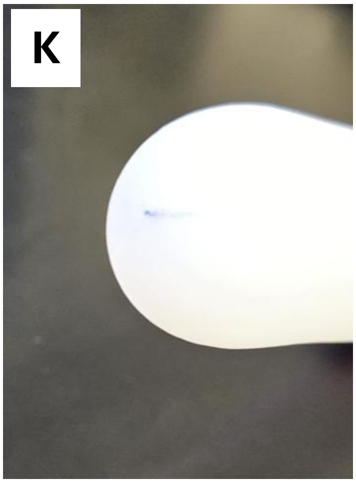

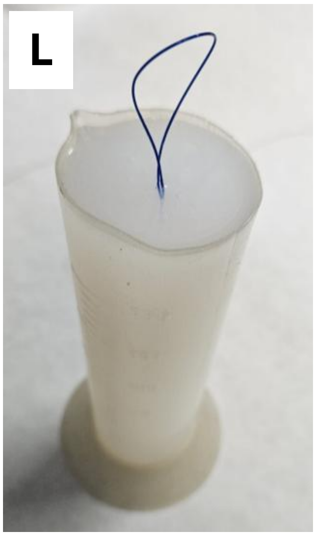

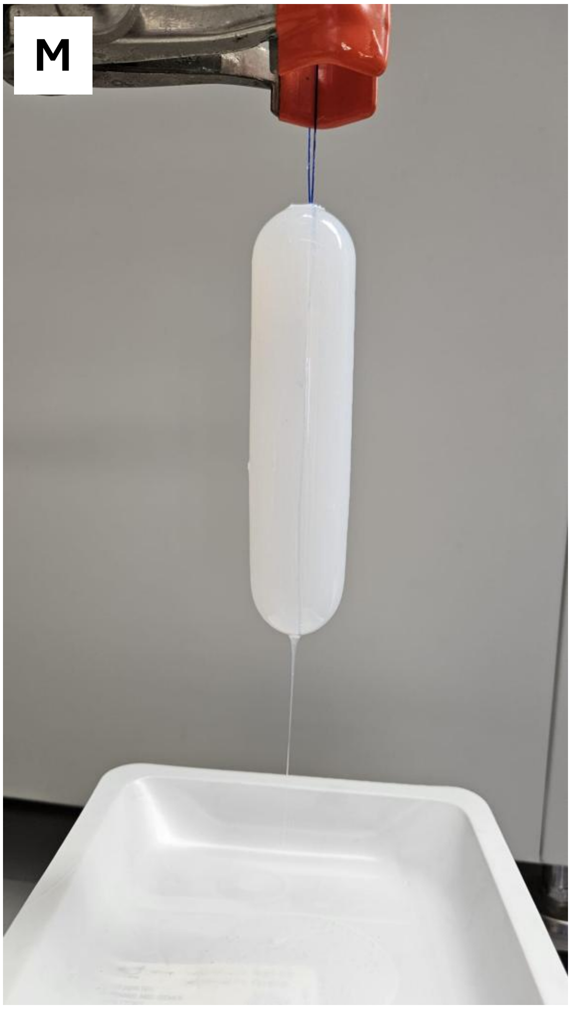

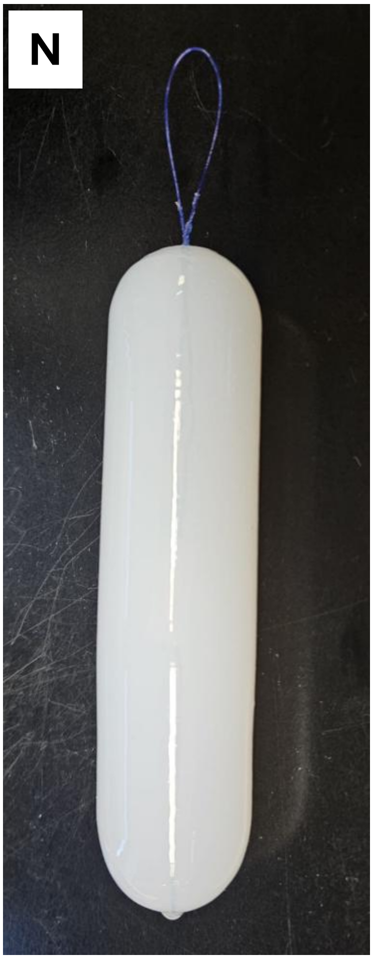

Supplement: Supplementary file 1 [file Data_Sheet_1.ZIP › Supplementary files/MOLD PREPARATION & FINAL COATING.pdf]
