# Supplementary material for: Development and use of a custom-designed vaginal dilator for post-surgical management in a congenital adrenal hyperplasia patient
Source: Front Med (Lausanne). 2026 May 25;13:1756295. doi: 10.3389/fmed.2026.1756295 (PMC13243258; doi:10.3389/fmed.2026.1756295)
Supplement: Supplementary file 1 [file Data_Sheet_1.ZIP › Supplementary files/Supplementary Table 1.pdf]

Instrument	Domain	What It Measures	Items	Domain Score Range	Scoring Method	Clinical Interpretation
FSFI	Desire	Sexual interest and desire	2	1.2–6.0	Sum × 0.6	Lower scores indicate reduced desire
FSFI	Arousal	Ability to become sexually aroused	4	0–6.0	Sum × 0.3	Lower scores reflect impaired arousal
FSFI	Lubrication	Vaginal lubrication quality and maintenance	4	0–6.0	Sum × 0.3	Lower scores indicate lubrication difficulties
FSFI	Orgasm	Ability to reach orgasm and satisfaction	3	0–6.0	Sum × 0.4	Lower scores indicate orgasmic dysfunction
FSFI	Satisfaction	Overall sexual satisfaction	3	0.8–6.0	Sum × 0.4	Lower scores reflect reduced satisfaction
FSFI	Pain	Pain during or after penetration	3	0–6.0	Sum × 0.4	Lower scores indicate more pain
FSFI Total	—	Global sexual function	19 items	2.0–36.0	Sum of all domains	**≤ 26.55 suggests sexual dysfunction**
CSFQ-F-C	Pleasure	Enjoyment of sexual activity	1	1–5	Single item	Lower scores indicate reduced pleasure
CSFQ-F-C	Desire/Frequency	Frequency of sexual thoughts and desire	2	2–10	Sum	Lower scores indicate reduced desire frequency
CSFQ-F-C	Desire/Interest	Level of sexual interest	3	3–15	Sum	Lower scores indicate reduced interest
CSFQ-F-C	Arousal/Excitement	Ability to become sexually excited	3	3–15	Sum	Lower scores indicate impaired arousal
CSFQ-F-C	Orgasm/Completion	Ability to reach orgasm	3	3–15	Sum	Lower scores indicate orgasmic difficulty
CSFQ-F-C Total	—	Global sexual functioning	12 items	19–75	Sum of all domains	**≤ 41 suggests sexual dysfunction**

Supplementary Table 1. Overview of the domains, structure, and scoring methods of the FSFI and CSFQ-F-C questionnaires.
The FSFI includes items scored from 0 or 1 to 5, depending on the question. Domain scores are obtained by summing the item values and applying a domain-specific multiplier, and the total score is the sum of all six domains. It assesses sexual function over the previous four weeks.
The CSFQ-F-C uses items scored from 1 to 5, with higher scores reflecting better functioning. Domain scores are calculated by adding the item values within each domain, and the total score is the sum of all domains. It evaluates sexual functioning over the previous month.
Detailed item lists for both instruments are available in the original publications by Rosen [14] and Clayton [15], in the manuscript References list.
